# FOXA1 Regulation Turns Benzamide HDACi Treatment Effect-Specific in BC, Promoting NIS Gene-Mediated Targeted Radioiodine Therapy

**DOI:** 10.1016/j.omto.2020.08.015

**Published:** 2020-08-29

**Authors:** Maitreyi Rathod, Madhura Kelkar, Snehal Valvi, Girish Salve, Abhijit De

**Affiliations:** 1 Molecular Functional Imaging Laboratory, ACTREC, Tata Memorial Centre, Navi Mumbai 410210, India; 2 Homi Bhabha National Institute, Anushakti Nagar, Mumbai 400094, India

**Keywords:** sodium iodide symporter, breast cancer, benzamide, HDAC inhibitor, transcriptional factor, radio-iodine therapy, FOXA1

## Abstract

Human sodium iodide symporter (*NIS*) gene mediated radio-ablation is a successful procedure in thyroid cancer clinics. In recent years, natural expression of NIS is reported in breast cancer (BC) cases but is yet to make its mark as a therapeutic procedure in BC clinics. A pre-exposure to histone deacetylase (HDAC) inhibitors to amplify endogenous NIS expression was attempted, but achieving cancer tissue-specific enhancement of NIS in patients is an important challenge to win. Here, for the first time, we show that a benzamide class of HDACi (bHDACi) can significantly induce *NIS* gene expression and function (p < 0.05) in BC cells with minimal off-target effects. Transcription factor (TF) profiler and promoter binding array reveals 22 TFs differentially activated by CI-994, of which FOXA1 is identified as a unique and positive regulator of NIS. Clonogenic assay shows reduced survival with bHDACi + ^131^I combination treatment. Further, AR-42 and MS-275 treatment shows enhanced NIS expression in an orthotopic breast tumor model. Combining bHDACi with 1 mCi ^131^I shows 40% drop in signal (p < 0.05), indicating enhanced radio-ablation effect. Cerenkov imaging revealed higher accumulation of ^131^I in MS-275-treated tumors. Thus, bHDACi-mediated selective enhancement ensuring minimal off-target effect is a step further toward using NIS as a therapeutic target for BC.

## Graphical Abstract

**Figure:**
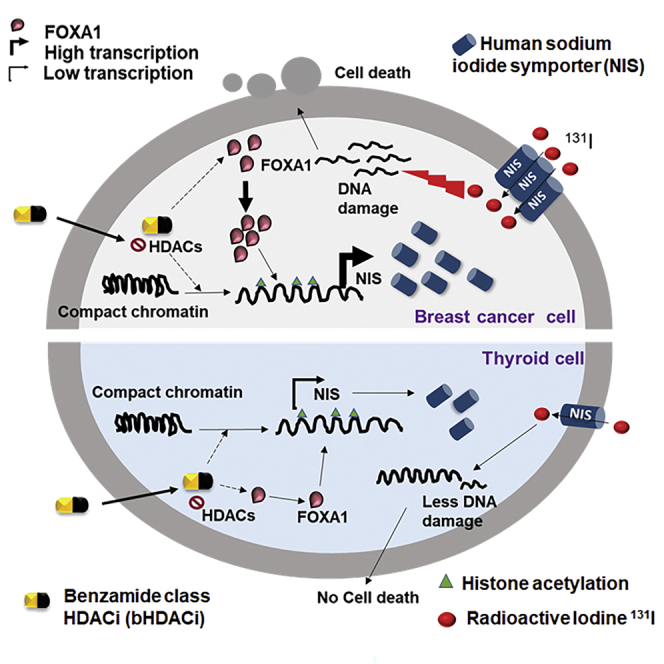

## Teaser

In this study, De and colleagues for the first time showed breast cancer tissue-specific transcriptional modulation of the hNIS gene, which can be achieved by using a benzamide class of HDAC inhibitors. Understanding the molecular basis of such cancer tissue-specific regulation may promote targeted radioiodine therapy application in BC patients with higher NIS expression.

## Introduction

The inherent heterogeneity of breast cancer (BC) is one of the major challenges for complete remission of the disease. The receptor-negative subtype (triple negative BC [TNBC]) still remains the most aggressive form of BC, with limited treatment options. Apart from chemotherapy and targeted therapies, radiation therapy also faces challenges of tumor relapse due to resistance.^1^ Gene therapy, particularly where the target gene is endogenous, can be considered for potential cancer treatment, and in that context, human sodium iodide symporter (*NIS*) gene is an attractive candidate. Adenoviral-mediated delivery of *NIS* gene in prostrate and colon cancer has been proven effective.^2^ Non-viral delivery methods like mesenchymal-stem-cell- or extracellular-vesicle-mediated delivery of *NIS* transgene have shown effective radio-iodine treatment in pre-clinical breast or hepatocellular carcinoma models, respectively.^3^^,^^4^ Although exogenous gene therapy has shown promising results in pre-clinical settings, efficient delivery to the target site, toxicity, ethical ramifications, etc. are major hurdles involved.^5^

Use of human NIS protein as an endogenous target is in practice for treatment of thyroid cancer (TC) patients using ^131^I.^6^ Physiologically, out of the vast number of solute carrier family of proteins, NIS is the sole candidate responsible for inward transportation of iodide ions in thyroid follicular cells, a requisite for thyroid hormone synthesis. NIS also functions in the mammary gland cells during lactation phase, and its aberrant overexpression in a majority of all BC subtype patients is also known.^7^ As an endogenously overexpressing target, addressing ethical, immunological, and toxicity-related issues are not relevant in this approach. However, to implement *NIS* gene-mediated targeted radio-iodine therapy protocol for BC patients, overcoming a few practical considerations are important for clinical translation. Clinical studies have shown that 70%–80% of the BC cases are NIS positive, of which around 30% cases show high intensity (2+/3+ score).^8^ Further, technetium-99 m uptake and scintigraphy imaging have shown that only 27% of all NIS-positive cases are functional.^9^ This fact can be co-related to the reports showing lack of membranous staining of NIS in a majority number of cases.^8^^,^^10^^,^^11^ Therefore, several groups around the world have attempted to reveal the mechanisms by which NIS expression can be modulated to improve on this treatment for BC. The modulators of NIS expression in case of TC are well characterized, where thyroid stimulating hormone (TSH) is primarily known for its control.^12^ All trans-retinoic acid (tRA) is known to induce NIS expression and function in BC through retinoic acid receptor/retinoid X receptor (RAR/RXR) receptors.^13^ Glucocorticoid agonists like dexamethasone and hydrocortisone, lactogenic hormones, insulin, insulin growth factor (IGF)-1, and IGF-2 are also reported as inducer of NIS expression in BC cells.^14^, ^15^, ^16^ Signaling pathways like mitogen-activated protein kinase (MAPK) can also contribute positively in regulating NIS function in BC.^17^ Pharmacological modulation of NIS by using HDAC inhibitor (HDACi) has also been proven efficacious for *NIS* gene-mediated radio-iodine therapy.^18^, ^19^, ^20^ As *NIS* gene expression and function is differently controlled in thyroid or breast cells,^21^ uptake of iodine in thyroid can be specifically blocked by T3, T4 saturation and/or combining with methimazole (MMI) treatment to prevent iodine organification in thyroid tissue.^22^ Here, we raised the question whether *NIS* gene expression can be specifically induced in BC cells, with minimal/no off target effect in thyroid cell? Currently, there is no such known procedure to boost NIS-mediated iodine uptake in a BC-specific manner with minimal or no effect on other relevant tissues. Therefore, this study provides evidence of such possibility by combining bHDACi drug pre-exposure with a follow-up ^131^I treatment. This study also explains the molecular basis of breast-tissue-specific transcriptional modulation of NIS and thus demonstrates a significant step forward toward clinical realization of *NIS* gene targeted radio-iodine therapy in BC.

## Results

### Benzamide Class of HDACi Can Induce *NIS* Gene Expression in BC Cells

Four drug inhibitors belonging to benzamide class of HDAC inhibitors (bHDACis) were selected in this study, all of which are at different stages of clinical trials as anticancer agents (Table S1). Since we aimed to use them as transcriptional modulators of NIS, a non-toxic dose (i.e., IC-30 equivalent or lower) was determined using BC cell lines MCF-7, ZR-75-1, and a TC cell line (ARO), i.e., 10 μM CI-994, 1 μM chidamide, 5 μM MS-275, and 500 nM AR-42 (Figures S1A–S1D).

**Figure 1:**
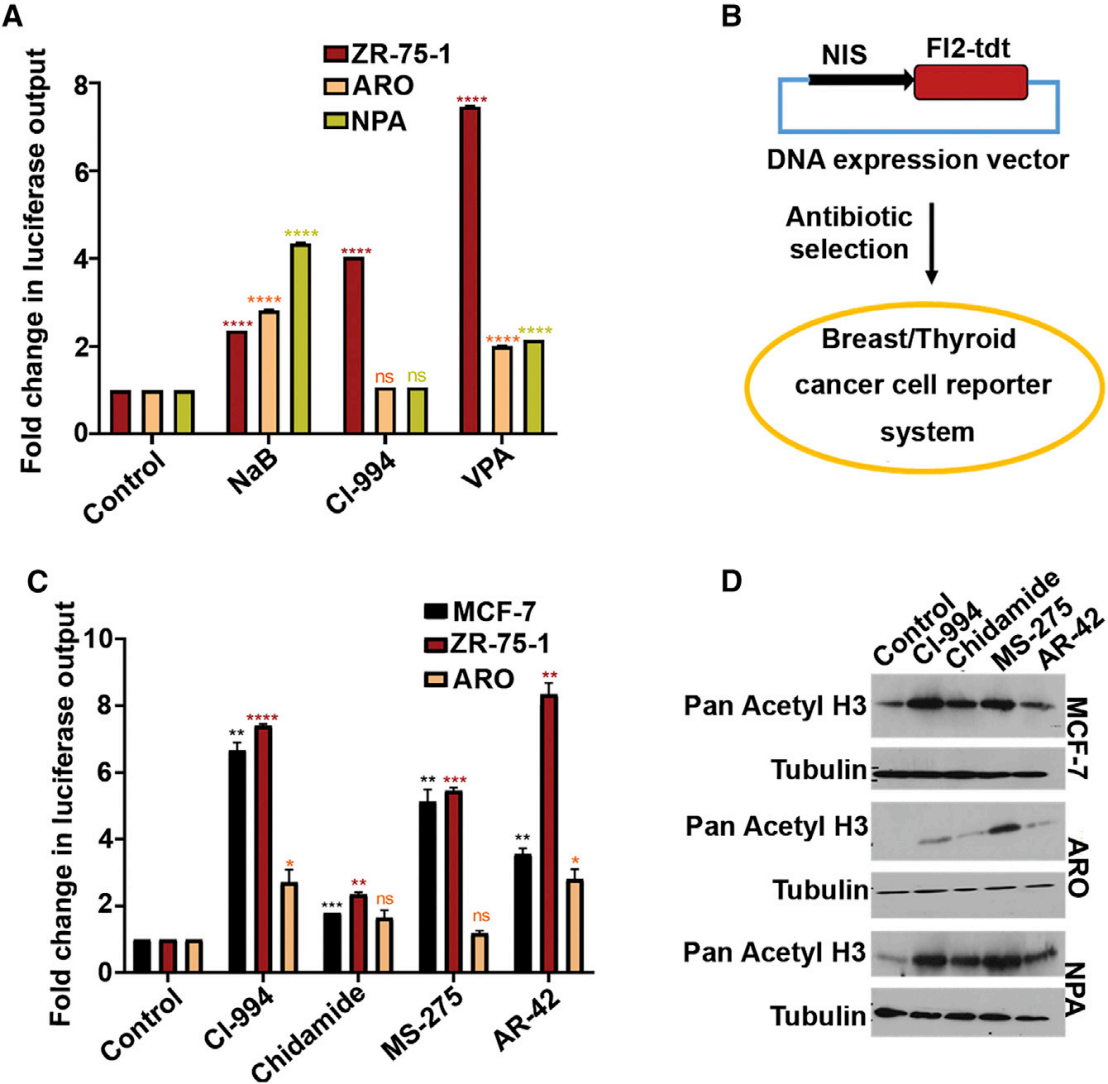
Benzamide Class of HDACi Enhances NIS Gene Expression in BC Cells (A) Graph showing fold difference in normalized NIS promoter driven luciferase reporter activity in non-benzamide (NaB, VPA) and benzamide (CI-994) drug-treated ZR-75-1, ARO, and NPA cells with respect to the untreated control. (B) Schematic explaining establishment procedure of pNIS-FL2.Turbo-overexpressing stable cell models using breast (MCF-7 and ZR-75-1) and TC (ARO) cell lines. (C) Bar graph showing fold differences of luciferase reporter activity in various benzamide drug-treated MCF-7, ZR-75-1, and ARO cells overexpressing pNIS-Fl2.Turbo construct. Error bar represents SEM; significance determined by t test. ∗∗p ≤ 0.01; ∗∗∗p ≤ 0.001. The color of the asterisk (∗) indicates the significance with their respective control. (D) Semiquantitative western blot showing increased pan acetylation of histone H3 in MCF-7, ARO, and NPA cells after treatment with various bHDACi drugs. Tubulin is used as loading control.

Thereafter, to study the effect of HDACi on NIS promoter activity, ZR-75-1 (BC) cells and ARO, NPA (TC) cells were transiently transfected with a NIS promoter-driven reporter plasmid. Non-benzamide classes of HDACi candidates like NaB and VPA show 3- to 7-fold increase of NIS promoter activity in both BC (ZR-75-1) and TC (ARO, NPA) lines, whereas CI-994, belonging to benzamide class HDACi, shows significant (p < 0.0001) gain of NIS promoter activity only in the ZR-75-1 cells (Figure 1A). Thereafter, to verify the differential modulatory effect of bHDACi on NIS promoter activity, we established MCF-7, ZR-75-1, and ARO cells stably overexpressing the same promoter-reporter expression plasmid (pNIS-FL2.Turbo) where the NIS promoter activity can be measured by luciferase expression (Figure 1B). Treatment of these engineered BC cells with bHDACi shows approximately 6- to 8-fold higher luciferase reporter expression (p ≤ 0.0001), except for chidamide drug, where about a 3-fold increase is recorded. In contrast, the same drug candidates show only 3-fold or less reporter activity (p = 0.1863) in the ARO cell line (Figure 1C). Increased acetylated histone H3 status confirms specific HDACi drug action in MCF-7, ARO, and NPA cells (Figure 1D).

**Figure 2:**
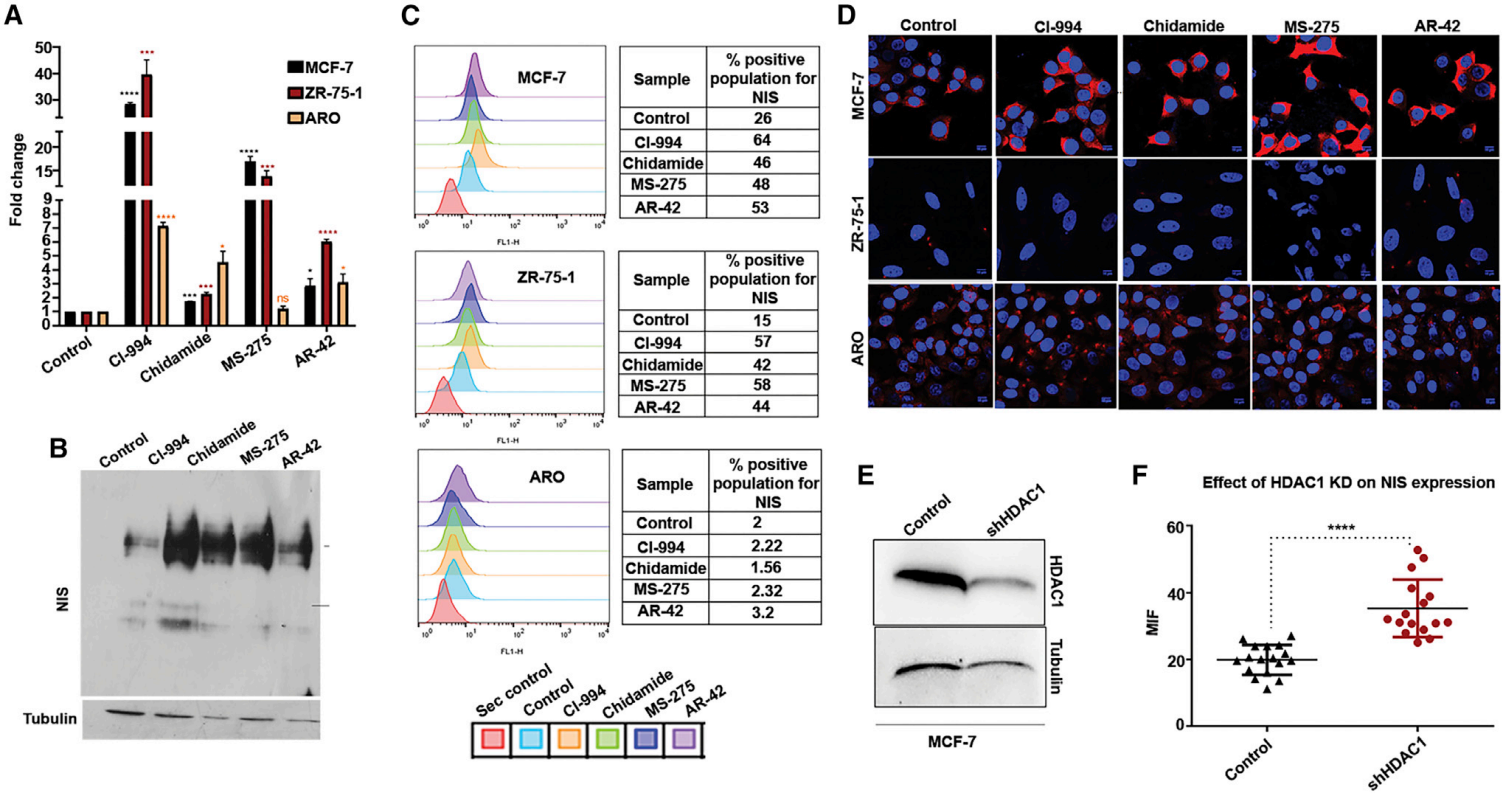
bHDACi Induces NIS Expression Specifically in BC Cells, but Not in Normal Breast or TC Cells (A) Graphs showing fold difference in NIS transcript levels for ZR-75-1, MCF-7, and ARO cell lines post-bHDACi treatment with respect to their untreated counterparts. Error bar represents SEM; significance determined by t test. ∗p ≤ 0.05; ∗∗∗p ≤ 0.001; ns, non-significant. (B) Semiquantitative western blot showing increased NIS expression (100-kDa band) post-bHDACi treatments for 48 h. (C) Data showing NIS-positive cell population after bHDACi treatment. Peaks that shift toward right on the *x* axis indicate increased FITC-positive populations. Table indicates percentage positive cell calculated from the histograms. (D) Immunofluorescence photomicrographs showing the NIS protein expression detected by using Dylight 633-labeled secondary antibody (red) in MCF-7, MCF-10A, and ARO cells. Blue indicates DAPI staining. Scale bar shows 10 μm distance. (E) HDAC1 protein expression in MCF-7 cells before and after knockdown of HDAC1. (F) Graph showing the MFI from individual control and HDAC1 knockdown cells (n = 30). ∗∗∗p ≤ 0.001.

Further, to estimate the bHDACi treatment effect directly on endogenous NIS transcription in BC or TC cell lines, quantitative RT-PCR analysis was done. Compared to untreated cells, BC cells treated with CI-994 or MS-275 show a highly significant (p < 0.001) increase of NIS transcript, whereas AR-42 and chidamide drug treatment shows either low or non-significant increase of NIS transcript, respectively (Figure 2A). Importantly, the fold increase observed in the BC cell types are drastically higher than the observed increment in the TC cell line, at least for CI-994 and MS-275. Thereafter, the effect of bHDACi on cellular NIS protein expression was monitored by performing immunofluorescence (IF) staining. Flow cytometry analysis of the antibody-stained cell population shows that after bHDACi treatment, only the BC cells, but not the TC cells, are capable of translating more NIS protein efficiently. Compared to untreated control, the NIS-positive population markedly increases after bHDACi treatment in MCF-7 and ZR-75-1 cells, whereas barely any change in NIS-positive fraction is recorded for the ARO cell line (Figure 2B). We have also checked if bHDACi treatment can enhance NIS protein expression in MCF-10A, an immortalized normal breast cell line. IF staining was performed in MCF-7, MCF-10A, and ARO cells, where only the MCF-7 cells show enhanced NIS expression after bHDACi treatment while MCF-10A or ARO cells display unaltered NIS expression (Figure 2C). A similar trend is also observed in additional BC and TC cell lines tested, i.e., ZR-75-1 and NPA, respectively (Figure S2A). The specificity of the bHDACi drug effect on BC cells is further demonstrated by using sodium butyrate (NaB), which is a short-chain fatty acid class of HDACi. NaB treatment shows enhanced NIS protein in ARO and NPA (Figure S2B). The induction of NIS protein levels in response to bHDACi is also verified by western blot, where bHDACi-treated MCF-7 cells show a dramatic increase in endogenous NIS expression (Figure 2D).

Further, most HDACi drugs act by inhibiting class 1 and/or class 2 HDAC enzymes, and MS-275 has a preference toward HDAC1. Therefore, we tested if the genetic knockdown of HDAC1 in MCF-7 cells can mimic the action of HDACi (Figure 2E). The knockdown of HDAC1 leads to a significant (p < 0.0001) increase in NIS expression, indicated by enhanced mean fluorescence intensity (MFI) of NIS (Figure 2F). NIS expression after HDAC1 knockdown was also quantitated by flow cytometry, where increased MFI is observed in HDAC1 knockdown conditions (Figures S3A and S3B). Thus, it appears that HDAC1 is a negative regulator of NIS expression, at least in MCF-7 cells.

### bHDACi-Induced NIS Function Can Deliberate Higher ^131^I-Mediated Cell Death

**Figure 3:**
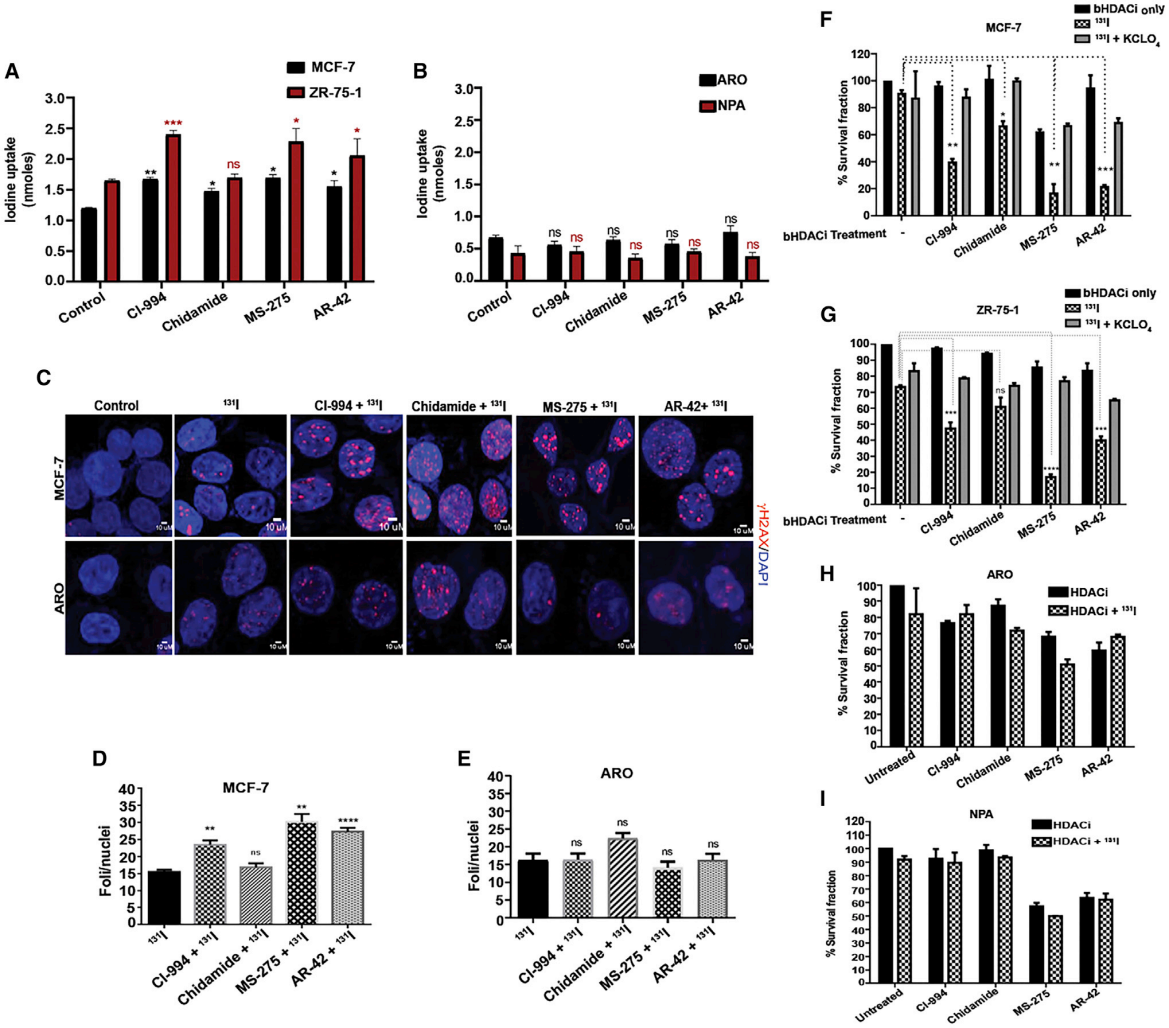
bHDACi Treatment Elevates the Function of NIS, thus Leading to Enhanced ^131^I -Mediated Cell Death in BC (A and B) Graphs showing the amount of iodine uptake (nmoles) in MCF-7, ZR-75-1 and ARO, NPA cells, respectively. Error bars indicate SEM. (C) γH2AX staining (red), indicating DNA damage response in MCF-7 and ARO cells after ^131^I treatment. Blue indicates nucleus staining using DAPI. Scale bar, 10 μm distance. (D and E) Graphs showing the quantification of the average number of γH2AX foci per nucleus, measured from at least 50 cells in MCF-7 and ARO cells, respectively. Error bars indicate SEM. (F and G) Graphs showing the survival fraction of MCF-7 and ZR-75-1 cells, respectively, after ^131^I treatment in the presence or absence of bHDACi treatment. 30 μM KCLO_4_ is used as a NIS-specific blocking agent for iodine transport. (H and I) Graphs represent results of similar experiment as in (F) and (G) for TC cell lines ARO and NPA, respectively. Error bar, SEM (∗∗p ≤ 0.01; ∗p ≤ 0.05).

To assess the functional relevance of elevated NIS protein in cell line models, we performed iodine uptake assay with or without bHDACi treatment in BC and TC cells. Treatment of BC cell lines (MCF-7 and ZR-75-1) with the bHDACi drug candidates lead to significant (p ≤ 0.05) increase in iodine uptake (Figure 3A), whereas the TC cell lines (ARO and NPA) show non-significant change in the iodine uptake after treatment (Figure 3B). Since the uptake of iodine is higher in BC cells pre-treated with bHDACi, we challenged the cells with ^131^I. ^131^I is a known inducer of DNA double-strand breaks; thus, MCF-7 cells show a higher number of γH2AX foci under bHDACi and ^131^I combined treatment as compared to ^131^I alone (Figures 3C and 3D). However, bHDACi-treated ARO cells do not show significant change in the number of γH2AX foci after being exposed to ^131^I, indicating insufficient DNA damage (Figures 3C and 3E). Since MCF-7 treated with bHDACi shows induced DNA damage, it is expected that they will show reduced cell survival. Therefore, untreated and bHDACi drug-treated cells were exposed to equal amounts of ^131^I (50 μCi of ^131^I for MCF-7, ARO, and NPA cells and 100 μCi for ZR-75-1). Treatment of only ^131^I and bHDACi in BC cells (MCF-7 and ZR-75-1) show 90%–70% cell survival in a long-term clonogenic assay. However, when treatment was done for a low dose of (IC-30 equivalent) bHDACi and ^131^I, a significant reduction (p < 0.05) in the surviving fraction is observed. The effect was most profound under CI-994, MS-275, and AR-42 treatment for both MCF-7 and ZR-75-1 cell lines. CI-994 combination with ^131^I leads to around 60% cell death in BC cells, while MS-275 + ^131^I causes 80% cell death in both MCF-7 and ZR-75-1 cells. To show NIS-specific ^131^I uptake, we used potassium perchlorate (KCLO_4_) blocker, which could rescue the radio-ablative cell death, showing 70%–80% cell survival (Figures 3F and 3G). The TC cell lines ARO and NPA, treated with bHDACi and a combination of bHDACi + I^131^, show no change in cell death (Figures 3H and 3I), which goes in line with our earlier findings, showing no change in NIS function in TC after bHDACi treatment.

### Low-Dose MS-275 or AR-42 Drugs Can Boost NIS Expression *In Vivo*

**Figure 4:**
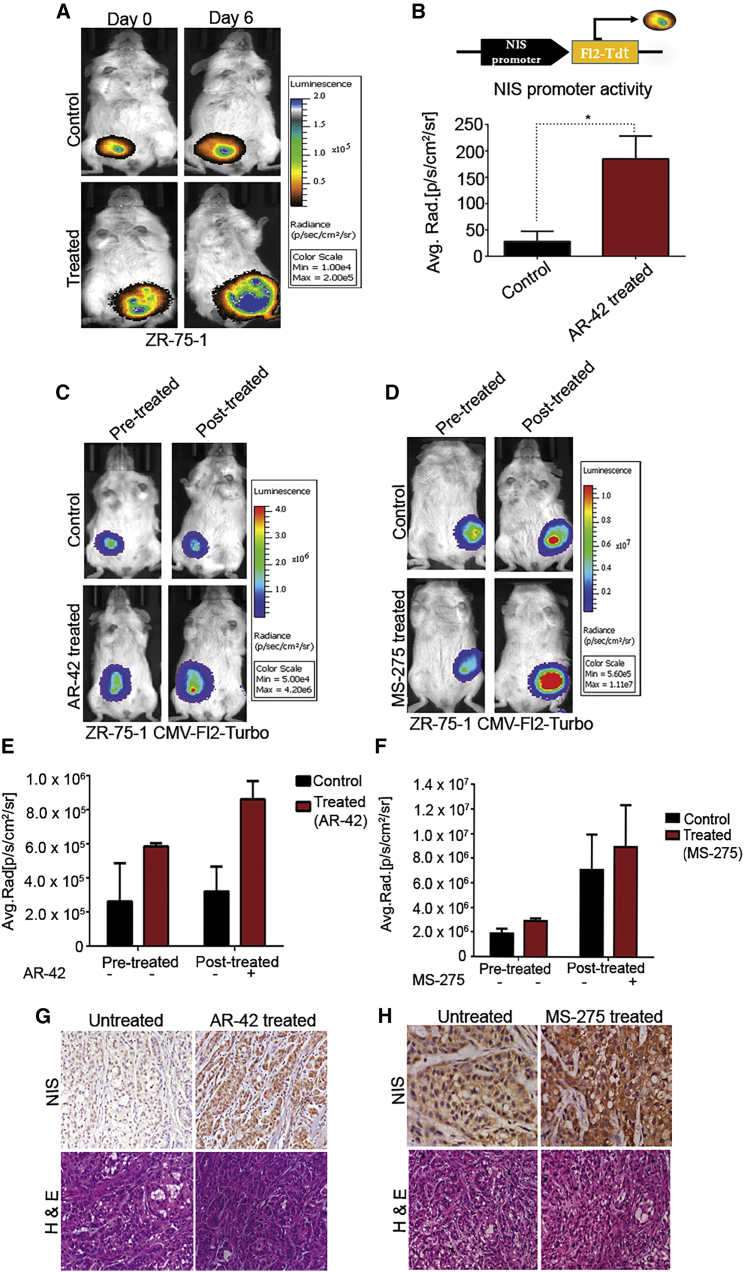
AR-42 and MS-275 Enhance the NIS Promoter Activity and Expression *In Vivo* (A) Bioluminescence (BLI) images of representative mouse with orthotopic ZR-75-1 NF tumors showing change in NIS promoter activity in untreated control and AR-42 drug-treated groups. (B) Graph showing BLI signal quantified from control and treated groups, normalized to the respective signals before treatment. Error bar indicates SEM (n = 2).∗p ≤ 0.05 calculated by Student’s t test. (C and D) BLI images of representative mouse, AR-42- or MS-275-treated groups, respectively, along with untreated control. Color scale bar indicates photon signal strength (photon/s/cm^2^/sr). (E and F) Graph showing quantified BLI signal from control and treated mice before and after AR-42 or MS-275 treatment, respectively. Error bar indicates SEM (n = 3). (G and H) Photomicrographs of NIS IHC performed on control and (G) AR-42- or (H) MS-275-treated tumors as marked. DAB staining (brown) and respective hematoxylin and eosin (H&E)-stained images are displayed.

NIS promoter-driven stable reporter system (ZR-75-1 NF) was used to generate orthotropic xenograft in immunodeficient mice for measurement of bHDACi effect on NIS transcriptional modulation *in vivo*. Bioluminescence (BLI) imaging shows treatment of 5 mg/kg AR-42 at every alternate day (three doses) can significantly increase (p ≤ 0.05) the reporter gene expression *in vivo* (Figures 4A and 4B). Simultaneous measurement of tumor volume indicates that signal increment is not due to change in tumor volume during the course of drug treatment (Figure S4). Further, we have also verified BLI signal output from ZR-75-1 tumors where the cells are engineered with CMV-Fl2.Turbo plasmid. Treatment with a low dose of AR-42 (5 mg/kg) and MS-275 (10 mg/kg) drugs show non-significant changes in reduced tumor growth (Figures 4C–4F). By performing immunohistochemistry (IHC) analysis for NIS on fixed tumor samples harvested from these mice, enhanced expression of NIS in treated tumor is confirmed, indicating that systemic use of low-dose drug can lead to increased expression of the endogenous NIS expression in tumors (Figures 4G and 4H).

**Figure 5:**
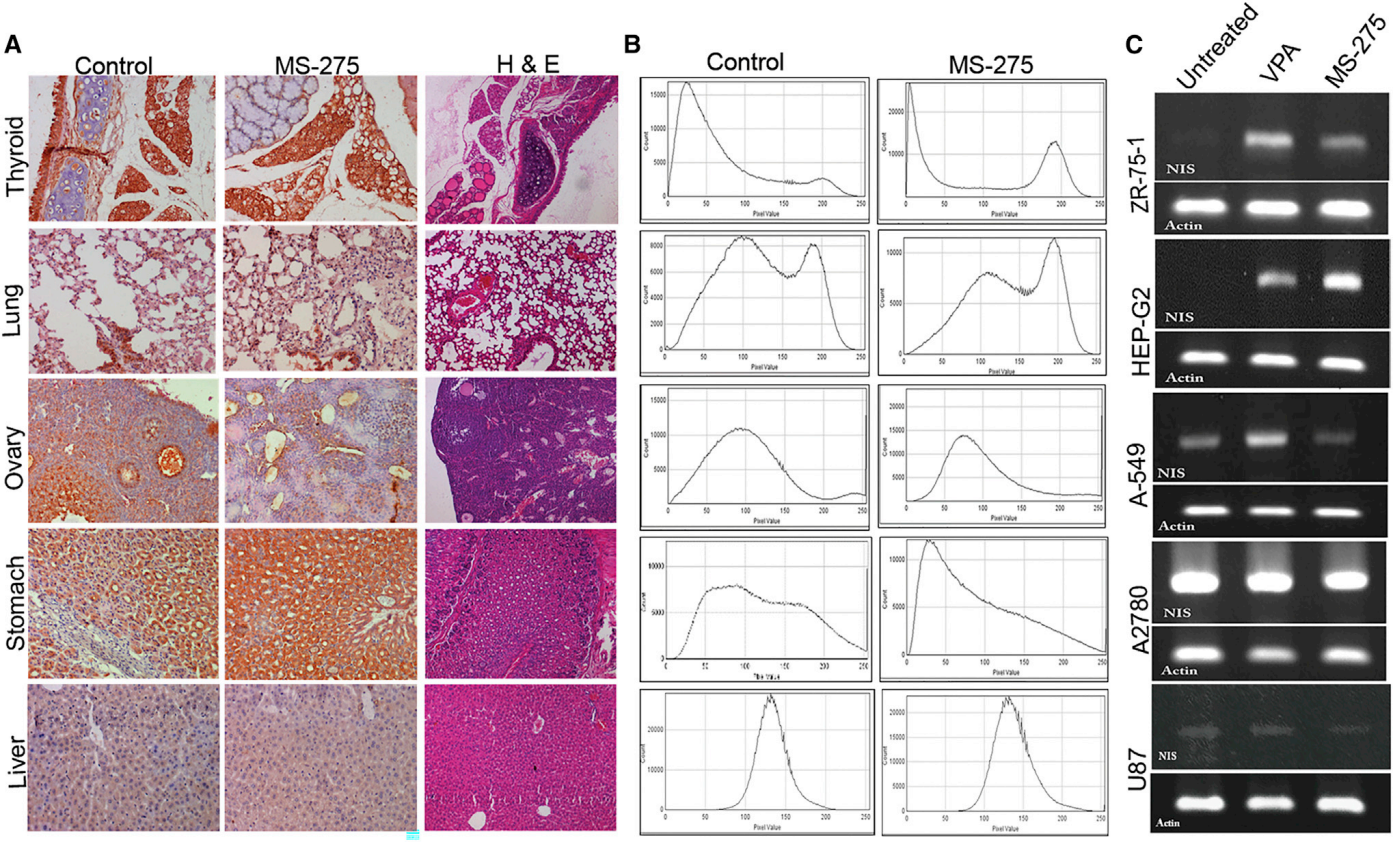
bHDACi Differentially Regulates NIS Expression across Organs *In Vivo* (A) Photomicrographs of NIS IHC performed on mouse thyroid, lung, ovary, stomach, and liver tissues harvested from untreated control or MS-275-treated mouse. DAB staining (brown) and respective H&E-stained images are displayed. (B) Histogram profiles of DAB-stained tissue sections showing NIS intensity profile obtained by using IHC Profiler program. (C) Images of gel picture showing amplified NIS transcripts as a measure of response to VPA and MS-275 drug treatment in various cancer cell lines, i.e., BC (ZR-75-1), lung cancer (A-549), hepatocellular carcinoma (HEPG2), ovarian cancer (A2780), and glioblastoma (U87). β-actin housekeeping gene is used as loading control.

Further, to judge the modulatory effect of bHDACi drug treatment on different organs *in vivo*, IHC staining analysis of NIS expression was done in different organs harvested from untreated and treated mice with MS-275 (10 mg/kg) drug. Organs that have a basal expression of NIS, like thyroid, lung, stomach, liver, and ovary, are primarily considered. Results indicate that MS-275 drug treatment can boost NIS expression in stomach. Other organs, such as thyroid, ovary, liver, and lung, did not show marked change in NIS staining (Figures 5A and 5B). Considering the main aspect of this study, NIS expression in thyroid remained unaffected after MS-275 treatment. Further, we also compared the modulatory effect of bHDACi candidate MS-275 with that of an aliphatic acid class of HDACi (valproic acid or VPA) in breast carcinoma (ZR-75-1), hepatocellular carcinoma (HEP-G2), lung (A-549), ovarian carcinoma (A2780), and glioblastoma (U87) cell lines (Figure 5C). Results indicate that both MS-275 and VPA induces NIS expression in breast and hepatocellular carcinoma cell lines, whereas MS-275-mediated induction of endogenous NIS expression is absent in lung cancer, ovarian cancer, and glioblastoma cell lines. Interestingly neither VPA nor MS-275 could augment NIS expression in glioblastoma (U87) and ovarian carcinoma (A2780) cell lines tested.

### bHDACi-Mediated Elevation of NIS Causes a Potential Radio-Ablation Effect in Orthotopic Breast Tumor Model

**Figure 6:**
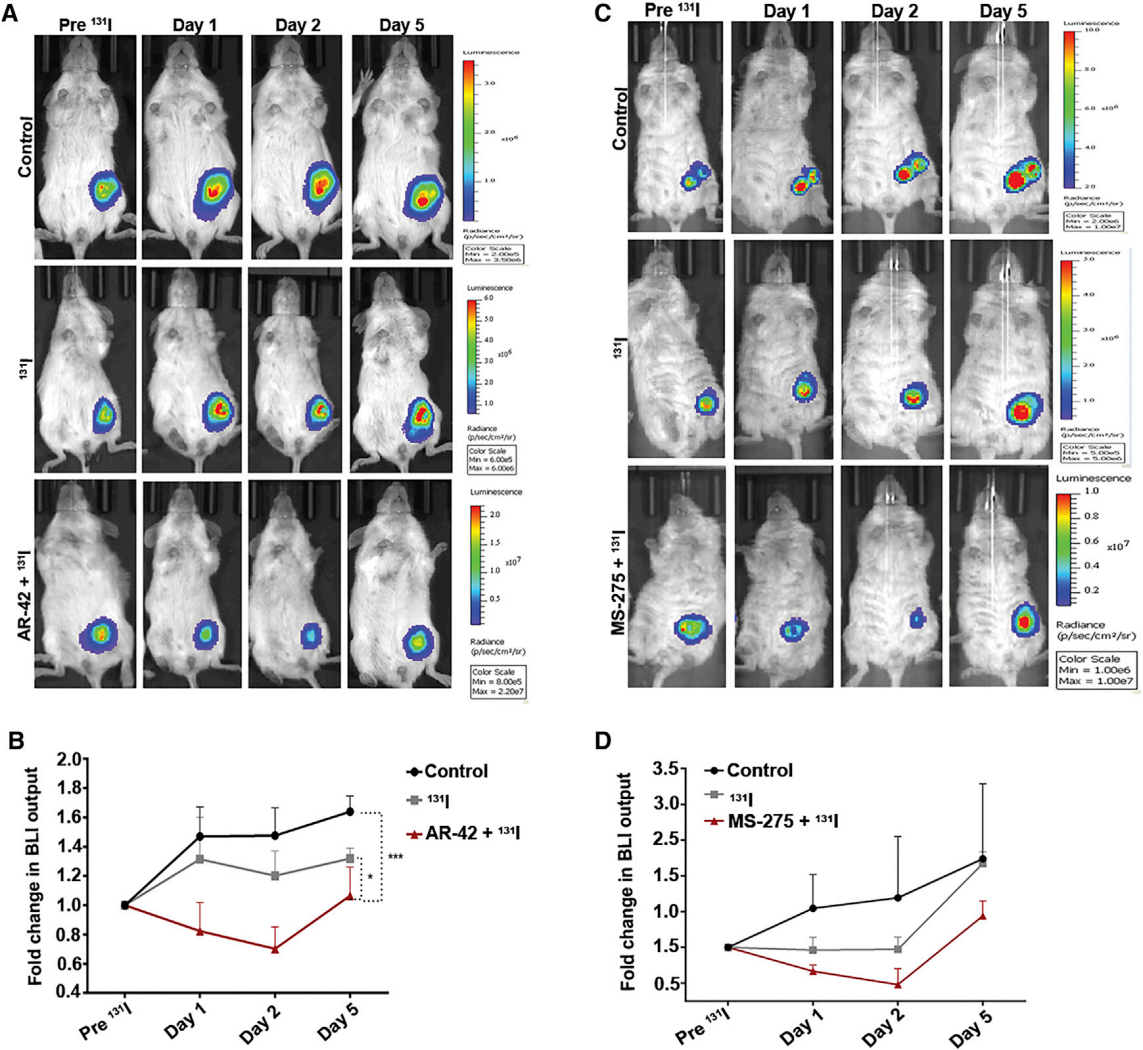
NIS-Mediated Radio-iodine Therapy Is Augmented in the Presence of AR-42 and MS-275 in BC (A and C) Panel of representative mouse images showing BLI signal from orthotopic ZR-75-1 (CMV-Fl2.Turbo) model. The change in BLI signal over 5 days after are shown for control, ^131^I only, and (A) AR-42 + ^131^I or (C) MS-275 + ^131^I groups as marked. (B and D) Graphs indicating the fold difference of quantified BLI signal for each with respect to the signals before ^131^I administration. Error bar indicates SEM calculated from three mice/group.

To demonstrate the efficacy of *NIS* gene-mediated radio-iodine therapy on *in vivo* orthotopic BC model, engineered ZR-75-1 cells overexpressing luciferase reporter (CMV-Fl2.Turbo) were used. Tumors from AR-42 + ^131^I-treated mice show a significant (p < 0.05) reduction in reporter signal, as compared to ^131^I-treated and -untreated control tumors. The BLI signal from AR-42 + ^131^I-treated tumors show regression till day 2 post-^131^I injection, and the remnant tumor mass starts re-growing after day 2; however, the tumors of control and ^131^I-treated mice continue to grow at every measured point (Figures 6A and 6B). Similarly, MS-275-treated mice also show a decrease in tumor growth after ^131^ I injection, up to day 2, after which the remnant tumour mass starts re-growing (Figures 6C and 6D). The distribution of ^131^I in tumors was measured by Cerenkov luminescence imaging. ^131^I luminescence counts are higher in tumors pre-treated with MS-275 as compared to tumors treated with only ^131^I (Figures S5A and S5B). The uptake of ^131^I in normal thyroid was estimated by gamma counter, indicating no significant difference in thyroidal uptake of ^131^I from bHDACi (CI-994) + ^131^I and ^131^I alone group (Figure S5C).

### FOXA1 Is a Positive Transcriptional Modulator of NIS Expression in BC

**Figure 7:**
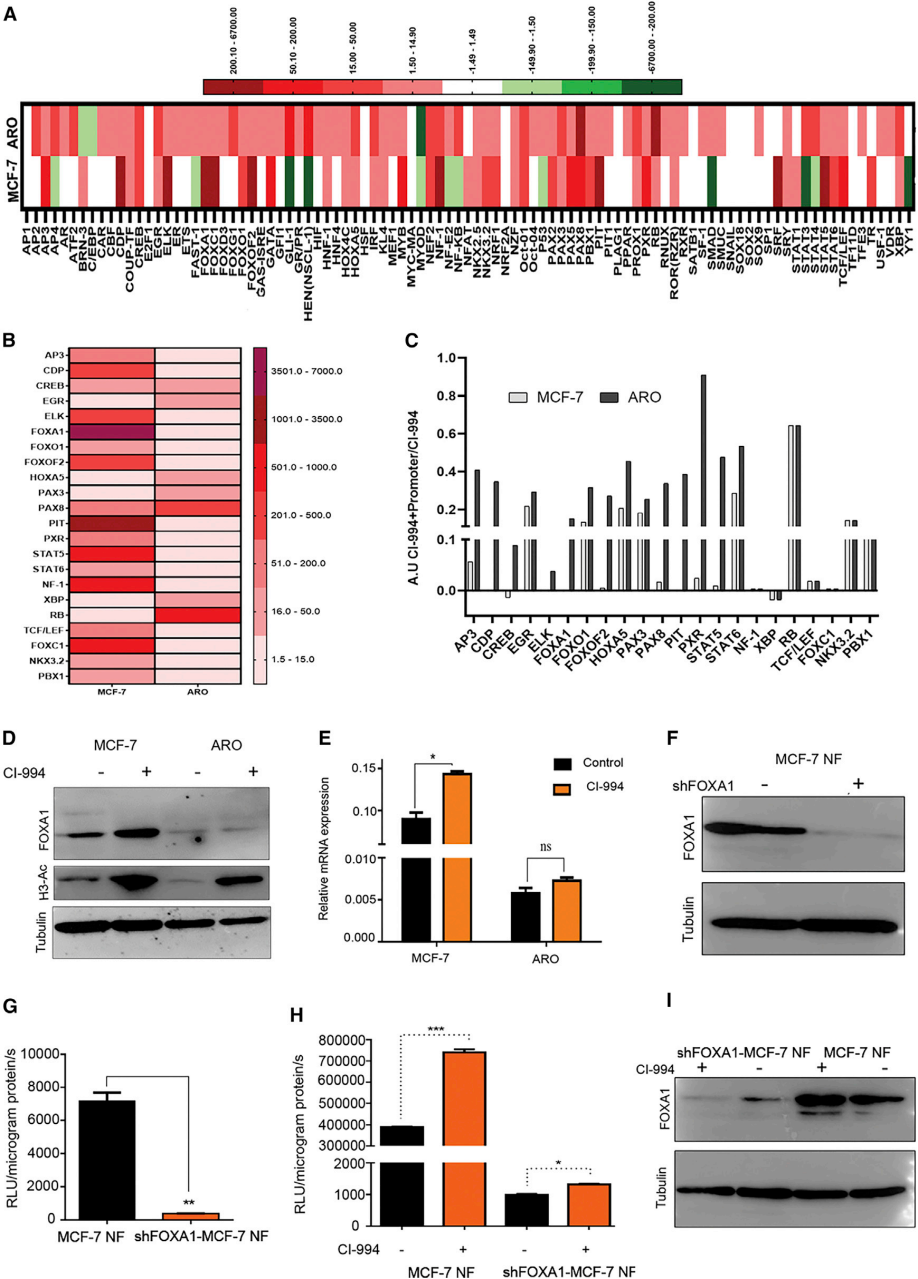
FOXA1 Positively Modulates NIS Expression in BC (A) Heatmap showing the activation/inhibition of 96 different TFs post-CI-994 treatment of MCF-7 and ARO cell lines. TF showing fold difference (CI-994-treated/-untreated) above 1.5 are considered as activated (red), while below −1.5-fold are negatively regulated (green) by CI-994. (B) Heatmap showing the differentially activated TFs in MCF-7 and ARO cell lines after CI-994 treatment. (C) Bar graph representing comparative binding efficiency of TFs between the MCF-7 and ARO cell line, where TF binding to the human NIS promoter was measured by promoter binding array after CI-994 treatment. (D) Semiquantitative western blot showing FOXA1 and H3-Ac expression in response to CI-994 treatment in MCF-7 and ARO cells. (E) Graph showing relative mRNA expression of FOXA1 with and without CI-994 treatment in MCF-7 and ARO cells. Error bar indicates SEM. ∗p ≤ 0.05; ns, non-significant, as derived from Student’s t test. (F) Semiquantitative western blot showing FOXA1 expression in control and shFOXA1 expressing MCF-7 NF cells. (G) Bar graph showing comparative luciferase reporter activity of shFOXA1-MCF-7 NF and MCF-7 NF cells. (H) Bar graph showing modulation of luciferase reporter activity of shFOXA1-MCF-7 NF and MCF-7 NF cells without or with CI-994 drug treatment. Error bar indicates SEM; ∗∗p ≤ 0.01. (I) Semiquantitative western blot showing FOXA1 expression in MCF-7 NF control and shFOXA1-MCF-7 NF cells with or without CI-994 treatment.

To identify the regulators (transcription factors, or TFs) enabling differential transcriptional modulation under bHDACi (CI-994) drug influence in BC, we performed a comparative assessment between MCF-7 and ARO cell lines by using a TF activation array involving 96 global TFs. As shown in Figure 7A, CI-994 treatment in the BC or TC cell line shows differential TF activation (> 1.5-fold, marked in red scale) or inhibition (< 1.5-fold, marked in green scale). Of the candidate TFs present in the array, 22 TFs show differential activation in response to CI-994 treatment in MCF-7 as compared to ARO. The TFs identified based on above parameters are AP3, CDF, CREB, EGR, ELK, FOXA1, FOXO1, FOXOF2, HOXA5, PAX3, PAX8, PIT, PXR, STAT5, STAT6, NF-1, FOXC1, TCF/LEF, NKX3.2, PBX1, RB, and XBP. The heatmap indicates activation status of these TFs after CI-994 treatment, normalized by untreated control (Figure 7B). As a confirmatory note, we see that known regulators like PAX8 are differentially activated in the TC cell line. In CI-994-treated MCF-7 cells, FOXA1 shows the highest differential activation. Further, these TFs also show binding to the NIS promoter, as was confirmed by promoter binding array performed by using human NIS promoter DNA for competitive binding in CI-994-treated cell samples. As the result suggests, all 22 TFs show specific binding to the unlabeled NIS promoter, indicated by 50% or lower signal drop (except for RB) in BC cell sample (Figure 7C). FOXA1 shows highest differential binding. Thereafter, a promo tool was used for bioinformatics analysis for putative TF binding on the human NIS promoter sequence, which also reveals that FOXA1 (or HNF-3α) has at least one putative binding site (Figure S6). Further, it was also verified that CI-994 treatment can enhance endogenous FOXA1 expression in the MCF-7 but not in the ARO cell line (Figure 7D). Increase in pan H3 acetylation after CI-994 treatment confirms equivalent drug activity in both cell types. Exclusively higher FOXA1 transcript expression (p ≤ 0.05) is also recorded in CI-994-treated MCF-7 cells (Figure 7E).

To demonstrate FOXA1 regulation of NIS expression, using the MCF-7 NF cell clone, we further established cells with stable shRNA mediated knockdown of FOXA1 (shFOXA1-MCF-7 NF; Figure 7F). FOXA1 knockdown shows a significant (p < 0.01) decrease in NIS promoter activity (Figure 7G) and NIS mRNA expression (Figure S7), indicating that FOXA1 is a positive modulator of NIS. We further tested if CI-994 treatment can enhance NIS promoter activity in shFOXA1-MCF-7 NF cell. Interestingly, we found that CI-994 treatment can’t substantially elevate NIS expression in FOXA1 knockdown cell, highlighting the pivotal role of this TF in controlling NIS expression in BC (Figure 7H). Further CI-994 treatment failed to enhance FOXA1 expression specifically in shFOXA1-MCF-7 NF as compared to basal MCF-7 NF cells (Figure 7I). Retrospective analysis from The Cancer Genome Atlas (TCGA) datasets reveals that the expression of FOXA1 is higher in BC patient cohort (METABRIC dataset, n = 2509) as compared to TC (Cell 2018 dataset, n = 498) patients (Figure S8). Additionally, FOXA1 amplifications were observed exclusively in BC cases, rendering the dependence of breast tumors to FOXA1.

## Discussion

Over the past two decades, a high abundance of *NIS* gene expression was reported in BC cases from many parts of the world.^7^, ^8^, ^9^, ^10^^,^^23^^,^^24^ Such reports have led to the enthusiastic proposition for its use as a therapeutic gene candidate for targeted radio-iodine therapy in BC-affected patients. NIS-mediated radio-ablation therapy is a promising approach in TC clinics. However, due to the clinical limitations pertaining to (1) its level of expression in BC at a much lower scale than in thyroid and (2) the safety concerns in terms of protecting the patient’s normal thyroid function, *NIS* gene therapy trials are not yet done in the clinic. By now, the major molecular regulators of NIS in thyroid or BC are fairly well understood.^17^^,^^22^^,^^25^, ^26^, ^27^ Our and others’ ability to boost NIS expression in cancer cells by using HDACi has shown promises to circumvent the first issue mentioned above.^18^, ^19^, ^20^ Our data showing the effect of stable knockdown of HDAC1 results in mediating significant enhancement of NIS expression in MCF-7 cells further brings additional prospect to this approach. But the safety issue that remains in systemic use of HDACi drug as a transcriptional modulator to enhance NIS expression is an expected global boost of endogenous NIS expression in multiple tissue types. Here, we find that benzamide-class HDACi can elevate the expression of NIS in a rather BC-cell-specific manner. Lack of transcriptional elevation in the normal breast cells (MCF-10A) and TC cells is of particular relevance to the problem.

Use of low-dose (IC-30 equivalent or lower) bHDACi is proposed here to achieve considerably higher amounts of NIS protein in BC cells. The levels achieved have also shown good radio-iodine therapeutic efficacy in cells. Additionally, all the bHDACis used here are in phase I or II clinical trials as anticancer agents for treatment of various solid tumors.^26^, ^27^, ^28^, ^29^ As our focus here is to test the use of these drugs as transcriptional modulators, minimally toxic but effective drug concentrations were determined in BC cells and used. Drug alone or ^131^I alone shows non-significant tumor growth retardation, while drug plus systemic ^131^I (single 1-mCi dose) combination shows a measurable reduction of tumor growth. Therefore, based on the pre-clinical non-invasive imaging experiments done, the bHDACi candidates such as MS-275 or AR-42 can be used for clinical trials on BC patients in the future. Based on the current findings, it can be implicated that, as per the practiced protocol using repeated doses of TSH and ^131^I in thyroid clinics, repeated use of bHDACi and ^131^I combination may also provide an improved outcome.

Further, we focused on identifying the molecular basis for the observed differential regulation of NIS in breast and TC cell types. By performing a TF binding array using bHDACi-treated MCF-7 and ARO cell samples, 22 differentially regulated TFs were identified. Of the candidates identified, Pax3, Pax8, and CREB are known regulators of NIS in thyroid cells, which provides the confidence in the results obtained. In BC cells, FOXA1 shows the highest binding to the human NIS promoter sequence in bHDACi-treated conditions. Interestingly, further confirmation on prospective roles of FOXA1 comes from the retrospective TCGA data analysis, which shows that FOXA1 expression is higher in BC patient as compared to TC patient samples. FOXA1 also shows amplification in BC patients and is absent in TC patients. The Human Protein Atlas also reports appreciably higher FOXA1 protein expression in BC tumor samples as compared to TC by IHC analysis. Therefore, we considered investigating the role played by FOXA1 on regulating *NIS* gene expression in BC cell under bHDACi drug treatment.

The MCF-7 cells with higher levels of basal FOXA1 expression (both transcript and protein) show a significant induction when treated with CI-994, but in the ARO cell line, we do not witness similar induction. Further, stable knockdown of FOXA1 in MCF-7 cells overexpressing human NIS promoter-driven firefly luciferase reporter (shFOXA1-MCF-7 NF) shows a significant loss of reporter gene expression as well as endogenous *NIS* gene expression. Thus, FOXA1 is a newly identified transcriptional modulator, which acts as a positive regulator of human *NIS* gene. Interestingly, by treating the shFOXA1-MCF-7 NF cells with CI-994, we find that in absence of this TF, the drug fails to induce endogenous *NIS* gene expression. As CI-994 can upregulate various other TFs in baseline MCF-7 cells, it is important to note here that the loss of FOXA1 alone downplaying the effect of CI-994 strongly indicates this candidate as a key modulator of NIS. Thus, we conclude that this study provides comprehensive evidence for re-purposing benzamide class HDACi in the context of *NIS* gene-mediated targeted radio-iodine therapy for BC treatment.

## Materials and Methods

### Reagents and Antibodies

MCF-7 and ZR-75-1 BC cells (ATCC) were maintained in RPMI-1640 media (Gibco, Invitrogen, USA); NPA and ARO (gifted by Mr. A. Chakraborty, BARC, India) were maintained in Iscove's modified dulbeccos medium (IMDM) (Gibco, Invitrogen, USA) containing 10% fetal bovine serum (FBS) and 0.075% gentamycin solution. HDACi MS-275 (1590-1), chidamide (2261), AR-42(2716-1), and CI-994 (1742-10) NaB and VPA were procured from Biovision. EXPOSE horseradish peroxidase (HRP)/3,3'-diaminobenzidine (DAB) detection IHC kit was from Abcam (ab80436), and MTT (3-[4, 5-dimethylthiazol-2-yl] c-2, 5-diphenyltetrazolium bromide) was from Sigma (USA). D-luciferin was procured from Biosynth Chemistry and Biology (L-8220), and luciferase assay system was from Promega (E4030). TF activation and promoter binding array (FA2002) was from Signosis (USA). FOXA1 primary antibody was from Abcam (ab-23738), H3 acetylation antibody (06-598) from Upstate (USA), human sodium iodide symporter antibody from Thermo Scientific (MA5-12308), alpha tubulin primary antibody from Sigma (T9026), γ H2Ax from Pierce Biotechnology (USA), anti-mouse HRP secondary antibody from Abcam (ab6728), goat anti-rabbit HRP antibody from Thermo Scientific (31460), and goat anti-mouse DyLight 633 secondary antibody from Thermo Scientific (35512). cDNA first strand synthesis kit was from Invitrogen (USA).

### Generation of NIS-Overexpressing Stable Cell Line and shFOXA1 Knockdown Stable Cell Model

MCF-7, ZR-75-1, and ARO cells were transfected with NIS promoter driving a fusion reporter Fluc2.TurboFP (pNIS-Fluc2.Turbo) plasmid DNA, using Lipofectamine 2000 reagent. Clones were selected with 500 μg/mL G418 antibiotic. Positive clones were verified by luciferase assay using LAR II substrate. The cells were constantly maintained under G418 selection until liquid N_2_ frozen stocks were made. The small hairpin RNA (shRNA) sequence of FOXA1 was designed and cloned into a Lentilox 3.7 vector. Lentiviruses containing the hFOXA1 DNA (GFP-positive) were made in HEK293FT cells. The virus were concentrated by high-speed ultra-centrifugation and transduced in MCF-7 NF cells. GFP-positive cells were selected over passages and amplified.

#### Luciferase Assay

The luciferase reporter assay was performed as per manufacturer’s recommended protocol. The relative light unit (RLU) was normalized with the respective protein concentrations of the lysates.

#### Immunoblotting

For immunoblots, cells were lysed using radioimmunoprecipitation assay buffer (RIPA) buffer containing protease inhibitor cocktail. Equal amounts of protein from control and transfected/treated cells were resolved in 7.5% SDS-PAGE gel and transferred onto a nitrocellulose membrane by semi-dry blotting apparatus. After blocking with 5% non-fat dry milk, membranes were probed with anti-human NIS antibody, anti-α-tubulin, and FOXA1. The blots were then probed with HRP-conjugated secondary antibodies and developed using a Chemidoc system.

#### IF and IHC Procedures

For IF experiments, cells were fixed with 4% paraformaldehyde for 10 min at 37°C. Blocking was done with 2% BSA. Primary antibody was incubated overnight at 4°C, followed by 1 h incubation with secondary DyLight 633 antibody. Fluorescence micrographs were captured using a Carl Zeiss LSM 780 confocal microscope. The magnification used was 63× objective, with a numerical aperture of 1.3 and pinhole restricted to 1 a.u. (1 AU = 0.7 μm). For IHC, tumors and organs from drug (MS-275, AR-42)-treated and control group were harvested and fixed in 10% formalin. For digital scoring of IHC slides, we used the IHC profiler plugin for ImageJ (software) developed by our group.^28^ IHC staining for NIS was performed as described in -Kelkar et al.^20^

#### Intracellular Staining of NIS by Flow Cytometry

Fluorescence-activated cell sorting (FACS) buffer was made by adding 0.01% sodium azide and 2% FBS in 1× PBS. 0.25% saponin for 15 min at room temperature was used for permeabilization of the cells. 1:70 dilution of primary NIS antibody was used, with an incubation of 45 min on ice, followed by anti-mouse fluorescein isothiocyanate (FITC) (Sigma, USA) secondary antibody for 45 min on ice.

#### Real-Time PCR

RNA was extracted using an RNeasy kit. cDNAs were synthesized using the first-strand cDNA synthesis kit. Quantitative real-time PCR was performed using Taqman probes for human NIS and glyceraldehyde 3-phosphate dehydrogenase (GAPDH) with assay IDs Hs00166567_m1 and Hs02758991_g1, respectively, on the 7900HT PCR cycler (Applied Biosystems, USA). Triplicate samples were run for each sample. The comparative ΔΔCt method was used to calculate relative gene expression.

#### Iodine Uptake Assay

Iodine uptake was done using the standardized non-radioactive assay method as described previously.^20^

#### TF Activation Profiling and NIS Promoter Binding Array

We performed a TF activation and promoter binding array for 96 different TFs as per the manufacturer’s guidelines. MCF-7 and ARO cells were treated with CI-994 for 48 h, and nuclear lysates from both control and treated cells were isolated using standard procedures and used as probe on the array plate. For analyzing promoter binding, the nuclear lysates were incubated with oligo-binding mix along with human NIS promoter DNA fragment. By comparing luminescence in presence or absence of competitor human NIS promoter, binding of various TFs were predicted.

#### In Vitro Clonogenic Assay

The *in vitro* clonogenic assay was performed as described by Mandell et al.^29^ MCF-7, ARO, and NPA were incubated with 50 μCi/mL ^131^I, and ZR-75-1 was incubated with 100 μCi/mL ^131^I for 5 h, after which the cells were washed with ice-cold Hank's balanced salt solution (HBSS) and seeded in a 6-well plate (1,000 cells/well).

#### In Vivo Optical Imaging

The experimental protocol was approved by the Institutional Animal Ethics Committee (IAEC) at ACTREC and performed in accordance with the guidelines for the Care and Use of the Laboratory Animals using ACTREC animal house and molecular imaging facilities. ZR-75-1 cells overexpressing Fluc2.TurboFP fusion gene (CMV-Fl2.Turbo) and ZR-75-1 cells overexpressing NIS promoter driving a fusion reporter Fluc2.TurboFP (pNIS-Fluc2.Turbo) were used to generate orthotopic BC models in female BALB/c SCID mice. The mice with ZR-75-1 CMV-Fl2.Turbo tumors were divided into three groups, i.e., the ^131^I group (intraperitoneal injection of 1 mCi Na-^131^I), AR-42, MS-275 + ^131^I group (three doses of 5 mg/kg of AR-42, 10 mg/kg of MS-275 every alternate day followed by 1 mCi Na-^131^I), and the control group (saline). After the tumor was initiated, all the groups received a daily intra-peritoneal injection of T4 (2 μg) + MMI (10 μg) for blocking the uptake of iodine in the thyroid glands for 21 days, following which AR-42 (5 mg/kg)/MS-275 (10 mg/kg) were given to AR-42/MS-275 + ^131^ I group mice at a total of three doses, every alternate day. 1 mCi ^131^I/mouse was then administered to ^131^I and AR-42/MS-275 + ^131^ I groups. BLI imaging was performed using IVIS-spectrum (Caliper Life Sciences) after intraperitoneal (i.p.) injection of 30 mg/mL of D-luciferin. Mice were anesthetized with isoflurane and placed in the imaging chamber with continuous 2% isoflurane administration via nasal cone. Data were analyzed using Living Image version 4.4 software.

#### Statistical Methods

All data are expressed as mean ± SE. Statistical significance was analyzed by Student’s t test using GraphPad Prism 6 software (GraphPad Software, La Jolla, CA, USA). p values ≤0.05 were considered statistically significant and CI was set at 95%. Multiple comparison was done using two-way ANOVA analysis for calculating the significance for *in vivo* ^131^I therapy pre-clinical study. Statistical analysis was done for a minimum of two independent biological repeats.

## Author Contributions

A.D. conceived the idea and designed the experiments; M.R., M.K., and G.S. performed the experiments; A.D. and M.R. analyzed the data and drafted the manuscript.

## Conflicts of Interest

The authors declare no competing interests.

## Supplemental Information

Document S1. Supplemental Materials and Methods, Table S1, and Figures S1–S8

Document S2. Article plus Supplemental Information

## References

[1] Jameel J.K., Rao V.S., Cawkwell L., Drew P.J. (2004). Radioresistance in carcinoma of the breast. Breast.

[2] Trujillo M.A., Oneal M.J., McDonough S., Qin R., Morris J.C. (2010). A probasin promoter, conditionally replicating adenovirus that expresses the sodium iodide symporter (NIS) for radiovirotherapy of prostate cancer. Gene Ther..

[3] Dwyer R.M., Ryan J., Havelin R.J., Morris J.C., Miller B.W., Liu Z., Flavin R., O’Flatharta C., Foley M.J., Barrett H.H. (2011). Mesenchymal Stem Cell-mediated delivery of the sodium iodide symporter supports radionuclide imaging and treatment of breast cancer. Stem Cells.

[4] Son S.H., Gangadaran P., Ahn B.C. (2019). A novel strategy of transferring NIS protein to cells using extracellular vesicles leads to increase in iodine uptake and cytotoxicity. Int. J. Nanomedicine.

[5] Birkeland A.C., Ludwig M.L., Spector M.E., Brenner J.C. (2016). The potential for tumor suppressor gene therapy in head and neck cancer. Discov. Med..

[6] Ahn B.C. (2012). Sodium iodide symporter for nuclear molecular imaging and gene therapy: from bedside to bench and back. Theranostics.

[7] Tazebay U.H., Wapnir I.L., Levy O., Dohan O., Zuckier L.S., Zhao Q.H., Deng H.F., Amenta P.S., Fineberg S., Pestell R.G., Carrasco N. (2000). The mammary gland iodide transporter is expressed during lactation and in breast cancer. Nat. Med..

[8] Chatterjee S., Malhotra R., Varghese F., Bukhari A.B., Patil A., Budrukkar A., Parmar V., Gupta S., De A. (2013). Quantitative immunohistochemical analysis reveals association between sodium iodide symporter and estrogen receptor expression in breast cancer. PLoS ONE.

[9] Moon D.H., Lee S.J., Park K.Y., Park K.K., Ahn S.H., Pai M.S., Chang H., Lee H.K., Ahn I.M. (2001). Correlation between 99mTc-pertechnetate uptakes and expressions of human sodium iodide symporter gene in breast tumor tissues. Nucl. Med. Biol..

[10] Renier C., Yao C., Goris M., Ghosh M., Katznelson L., Nowles K., Gambhir S.S., Wapnir I. (2009). Endogenous NIS expression in triple-negative breast cancers. Ann. Surg. Oncol..

[11] Rathod M., Chatterjee S., Dutta S., Kalraiya R., Bhattacharyya D., De A. (2019). Mannose glycosylation is an integral step for NIS localization and function in human breast cancer cells. J. Cell Sci..

[12] Lakshmanan A., Scarberry D., Shen D.H., Jhiang S.M. (2014). Modulation of sodium iodide symporter in thyroid cancer. Horm. Cancer.

[13] Tanosaki S., Ikezoe T., Heaney A., Said J.W., Dan K., Akashi M., Koeffler H.P. (2003). Effect of ligands of nuclear hormone receptors on sodium/iodide symporter expression and activity in breast cancer cells. Breast Cancer Res. Treat..

[14] Unterholzner S., Willhauck M.J., Cengic N., Schütz M., Göke B., Morris J.C., Spitzweg C. (2006). Dexamethasone stimulation of retinoic Acid-induced sodium iodide symporter expression and cytotoxicity of 131-I in breast cancer cells. J. Clin. Endocrinol. Metab..

[15] Kogai T., Kanamoto Y., Li A.I., Che L.H., Ohashi E., Taki K., Chandraratna R.A., Saito T., Brent G.A. (2005). Differential regulation of sodium/iodide symporter gene expression by nuclear receptor ligands in MCF-7 breast cancer cells. Endocrinology.

[16] Micali S., Bulotta S., Puppin C., Territo A., Navarra M., Bianchi G., Damante G., Filetti S., Russo D. (2014). Sodium iodide symporter (NIS) in extrathyroidal malignancies: focus on breast and urological cancer. BMC Cancer.

[17] Jung K.H., Paik J.Y., Ko B.H., Lee K.H. (2008). Mitogen-activated protein kinase signaling enhances sodium iodide symporter function and efficacy of radioiodide therapy in nonthyroidal cancer cells. J. Nucl. Med..

[18] Fortunati N., Catalano M.G., Marano F., Mugoni V., Pugliese M., Bosco O., Mainini F., Boccuzzi G. (2010). The pan-DAC inhibitor LBH589 is a multi-functional agent in breast cancer cells: cytotoxic drug and inducer of sodium-iodide symporter (NIS). Breast Cancer Res. Treat..

[19] Kitazono M., Robey R., Zhan Z., Sarlis N.J., Skarulis M.C., Aikou T., Bates S., Fojo T. (2001). Low concentrations of the histone deacetylase inhibitor, depsipeptide (FR901228), increase expression of the Na(+)/I(-) symporter and iodine accumulation in poorly differentiated thyroid carcinoma cells. J. Clin. Endocrinol. Metab..

[20] Kelkar M.G., Senthilkumar K., Jadhav S., Gupta S., Ahn B.C., De A. (2016). Enhancement of human sodium iodide symporter gene therapy for breast cancer by HDAC inhibitor mediated transcriptional modulation. Sci. Rep..

[21] Dutta S., De A. (2015). Cancer gene therapy: Prospects of using human sodium iodide symporter gene in non-thyroidal cancer. Biomed. Res. J..

[22] Kogai T., Brent G.A. (2012). The sodium iodide symporter (NIS): regulation and approaches to targeting for cancer therapeutics. Pharmacol. Ther..

[23] Wapnir I.L., van de Rijn M., Nowels K., Amenta P.S., Walton K., Montgomery K., Greco R.S., Dohán O., Carrasco N. (2003). Immunohistochemical profile of the sodium/iodide symporter in thyroid, breast, and other carcinomas using high density tissue microarrays and conventional sections. J. Clin. Endocrinol. Metab..

[24] Wapnir I.L., Goris M., Yudd A., Dohan O., Adelman D., Nowels K., Carrasco N. (2004). The Na+/I– symporter mediates iodide uptake in breast cancer metastases and can be selectively down-regulated in the thyroid. Clin. Cancer Res..

[25] Arturi F., Ferretti E., Presta I., Mattei T., Scipioni A., Scarpelli D., Bruno R., Lacroix L., Tosi E., Gulino A. (2005). Regulation of iodide uptake and sodium/iodide symporter expression in the mcf-7 human breast cancer cell line. J. Clin. Endocrinol. Metab..

[26] Beyer S., Lakshmanan A., Liu Y.Y., Zhang X., Wapnir I., Smolenski A., Jhiang S. (2011). KT5823 differentially modulates sodium iodide symporter expression, activity, and glycosylation between thyroid and breast cancer cells. Endocrinology.

[27] Dentice M., Luongo C., Elefante A., Romino R., Ambrosio R., Vitale M., Rossi G., Fenzi G., Salvatore D. (2004). Transcription factor Nkx-2.5 induces sodium/iodide symporter gene expression and participates in retinoic acid- and lactation-induced transcription in mammary cells. Mol. Cell. Biol..

[28] Varghese F., Bukhari A.B., Malhotra R., De A. (2014). IHC Profiler: an open source plugin for the quantitative evaluation and automated scoring of immunohistochemistry images of human tissue samples. PLoS ONE.

[29] Mandell R.B., Mandell L.Z., Link C.J. (1999). Radioisotope concentrator gene therapy using the sodium/iodide symporter gene. Cancer Res..

