# Supplementary material for: FOXA1 Regulation Turns Benzamide HDACi Treatment Effect-Specific in BC, Promoting NIS Gene-Mediated Targeted Radioiodine Therapy
Source: Mol Ther Oncolytics. 2020 Aug 29;19:93–104. doi: 10.1016/j.omto.2020.08.015 (PMC7554325; doi:10.1016/j.omto.2020.08.015)
Supplement: Document S1. Supplemental Materials and Methods, Table S1, and Figures S1–S8 [file mmc1.pdf]

OMTO, Volume *19*

Supplemental Information

FOXA1 Regulation Turns Benzamide HDACi Treatment Effect-Specific in BC, Promoting NIS Gene-Mediated Targeted Radioiodine Therapy

Maitreyi Rathod, Madhura Kelkar, Snehal Valvi, Girish Salve, and Abhijit De

Supplementary methods:

MTT cell toxicity assay:

To evaluate the IC-20, IC-30 concentrations of bHDACi, MCF-7, ZR-75-1 and ARO cells were seeded at a density of 5×10^3, in 96 well plates (Corning, USA), in triplicates. Cells were exposed to different concentrations of bHDACi for 48 hours. Cell viability was assessed using 10µl of 5mg/ml MTT (3-[4,5-dimethylthiazol-2-yl]c-2,5-diphenyltetrazolium bromide) (Sigma, USA) reagent, and absorbance measured at 570nm and 630nm.

Measurement of ^{131}I uptake by Gamma Counter:

Mice from CI-994 treated and untreated groups were injected with 1mCi ^{131}I. Day 1, day 3 and day 7 post ^{131}I injection, the mice were dissected and the thyroid gland was isolated. The uptake of ^{131}I from thyroid glands of CI-994+^{131}I and ^{131}I alone groups, was measured by HIDEX-AMG Gamma counter.

Cerenkov Luminescence Imaging:

CLI was performed using IVIS spectrum system. For *in vivo* Cerenkov imaging, animals in the ^{131}I alone or AR-42/MS-275+^{131}I groups were injected with 1mCi of ^{131}I intraperitoneally. Animals were placed in a light-tight chamber under isoflurane anaesthesia, and the luminescence scan was taken in open filter, F/Stop 1, subject height 1.5 cm.

Bioinformatics analysis:

FOXA1 expression analysis from breast and thyroid cancer patient samples was done by cBioPortal using METABRIC data set, n=2509 for breast cancer cohort and Cell 2018 dataset, n=498 for thyroid cancer cohort [2-4].

References:

1. West, A.C. and R.W. Johnstone, *New and emerging HDAC inhibitors for cancer treatment.* J Clin Invest, 2014. **124**(1): p. 30-9.
2. Cerami, E., et al., *The cBio cancer genomics portal: an open platform for exploring multidimensional cancer genomics data.* Cancer Discov, 2012. **2**(5): p. 401-4.
3. Pereira, B., et al., *The somatic mutation profiles of 2,433 breast cancers refines their genomic and transcriptomic landscapes.* Nat Commun, 2016. **7**: p. 11479.
4. Hoadley, K.A., et al., *Cell-of-Origin Patterns Dominate the Molecular Classification of 10,000 Tumors from 33 Types of Cancer.* Cell, 2018. **173**(2): p. 291-304 e6.

Supplementary table 1 (ST1): Table showing the different benzamide class HDAC inhibitors selected for the study **[1]**

**Group**	**Candidate**	**HDAC targets**	**Clinical Trial**	**Type of malignancy**
Benzamide	CI-994	Class 1 HDAC	Phase 2	Pancreatic cancer
	Chidamide	Class1,2 HDAC	Phase 2	Solid tumours, lymphoma
	MS-275	Class 1 (HDAC1,3)	Phase 2/1	Melanoma, Refractory solid tumours and lymphoma
	AR-42	Class1,2 HDAC	Phase 1	Haematological malignancies

Supplementary Figures

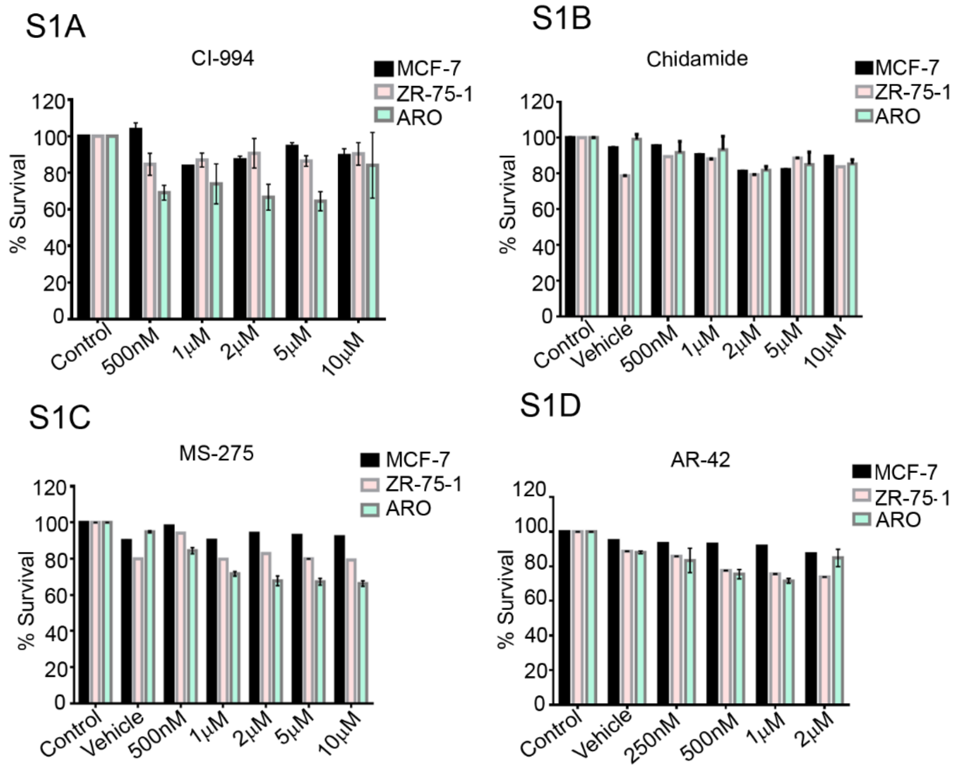

Supplementary Figure 1: **Benzamide class HDACi are non-toxic to MCF-7, ZR-75-1 and ARO cells**. ***S1A-D****: Graphs showing the cytotoxicity profile of CI-994, Chidamide, MS-275 and AR-42 at different concentrations in MCF-7, ZR-75-1 and ARO cell lines. Y-axis shows percent survival fraction with respect to their untreated controls.*

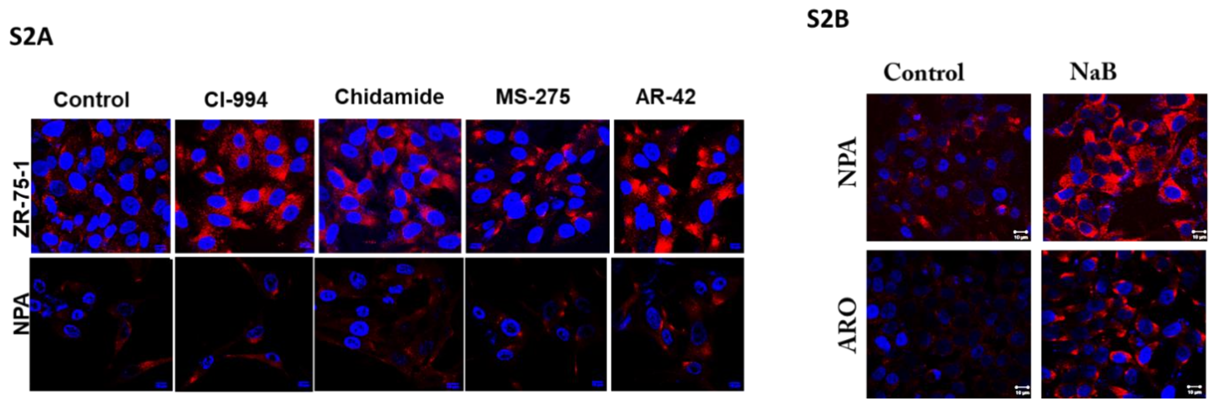

Supplementary Figure 2: **bHDACi enhance NIS expression in MCF-7 cells and sodium butyrate induces NIS in TC cells.** ***S2A****: Immunofluorescence images showing NIS (red) expression in ZR-75-1 and NPA cells in response to bHDACi treatments. Blue indicates nucleus stained by DAPI.* ***S2B:*** *Immunofluorescence images showing enhanced NIS expression in ARO, NPA cells after sodium butyrate (NaB) treatment.*

S3A

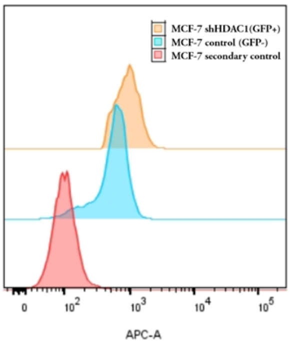

S3B

Sample	Mean intensity
MCF-7 control	697
MCF-7 shHDAC1	1120

Supplementary Figure 3: **HDAC1 is a negative regulator of NIS expression in breast cancer.** ***S3A****: Flow cytometry graph showing enhanced NIS expression after HDAC1 knockdown (MCF-7 shHDAC1-GFP+ve) which is the orange peak. The blue peak shows parental MCF-7 cells and red peak shows secondary control.* ***S3B:*** *Table showing the difference in the mean fluorescence intensity of NIS (APC channel) as quantified by flow cytometry.*

S4A

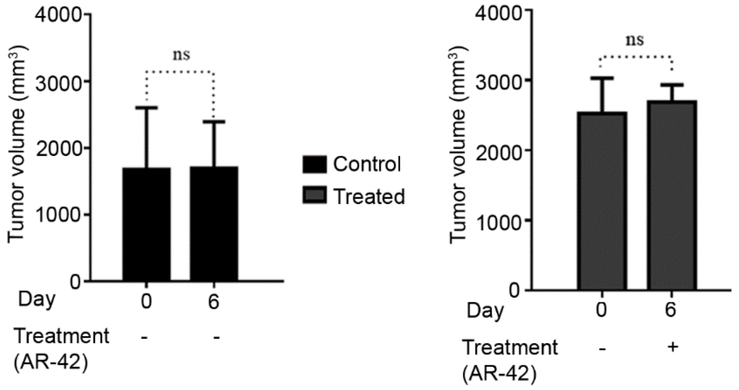

Supplementary Figure 4: **Sub optimal dose of AR-42 treatment has no effect on tumour volume.** ***S4:*** *Charts showing tumour volume from control and treated mice. Tumour volumes were measured on day 0 (pre-treatment) and day 6 (post-treatment), as indicated on x-axis. ns stands for non-significant.*

S5A

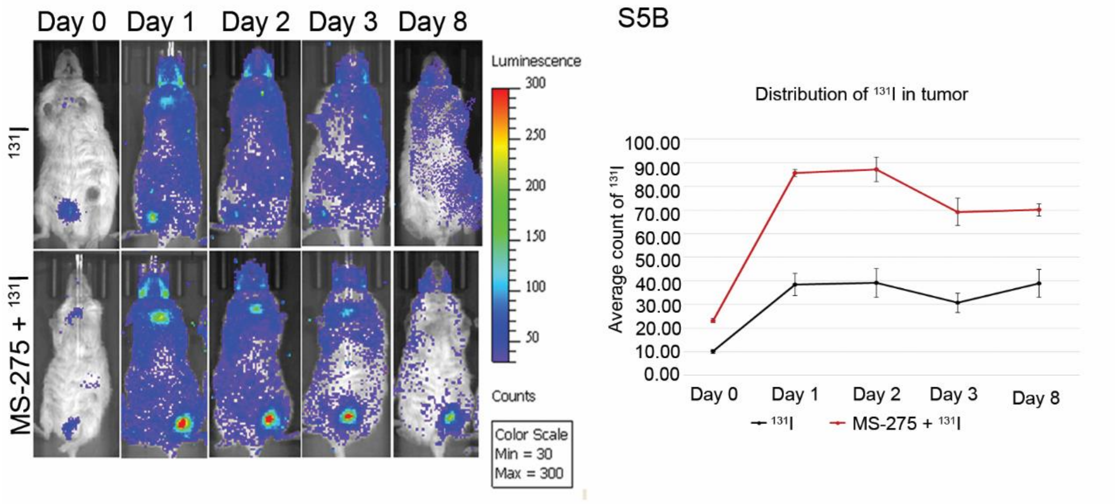

S5C

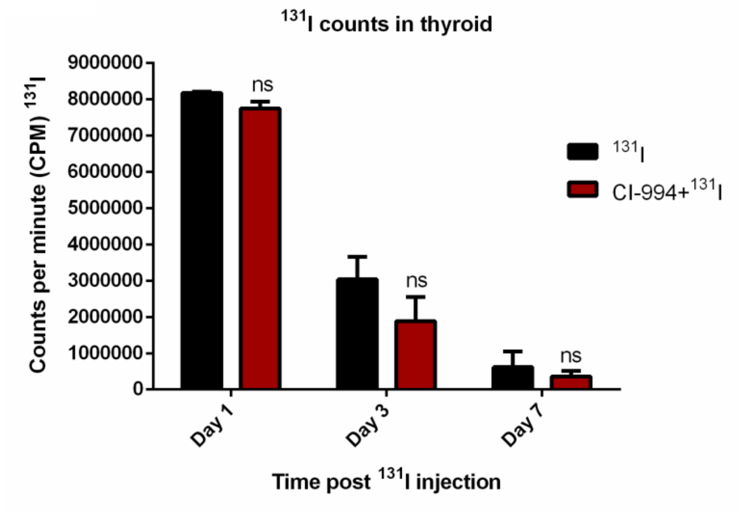

Supplementary Figure 5: **Cerenkov luminescence imaging showing higher accumulation of ^{131}I in MS-275+ ^{131}I group.** ***S5A:*** *Mice images showing Cerenkov luminescence after ^{131} I injection in ^{131}I and MS-275+^{131}I groups. Scale bar shows the counts of Cerenkov luminescence output.* ***S5B:*** *Chart depicting the distribution of ^{131}I in tumour.* ***S5C:*** *Graph indicating in vivo uptake of ^{131}I in thyroid gland of ^{131}I and CI-994+^{131}I groups. ns stands for non-significant.*

S6

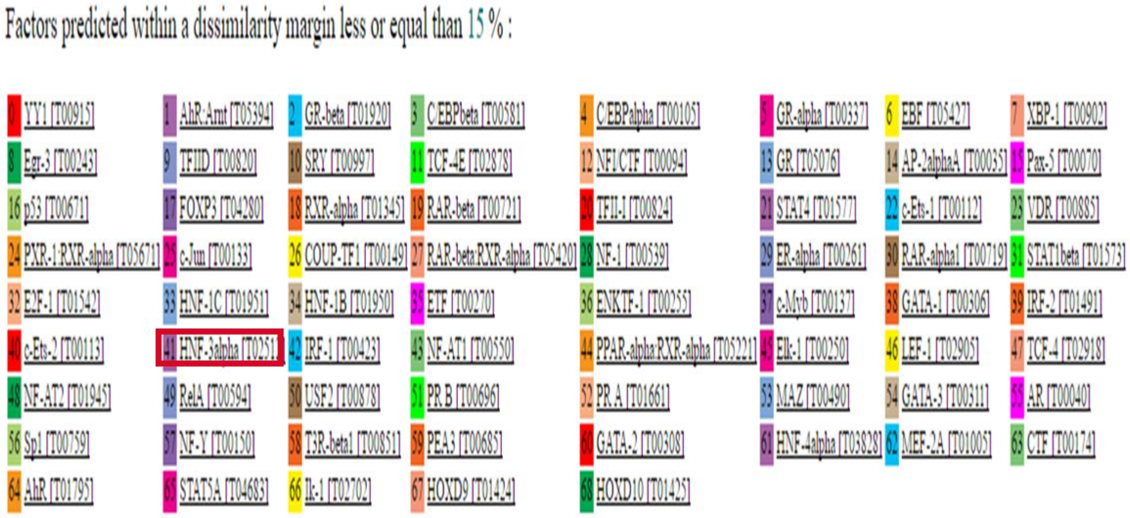

Supplementary Figure 6: **HNF3 alpha (FOXA1) has putative binding sites on NIS promoter.** *Image showing the putative transcription factors that bind to NIS promoter with 15% dissimilarity range, as predicted by promo software. HNF3-alpha (FOXA1) is highlighted in red box.*

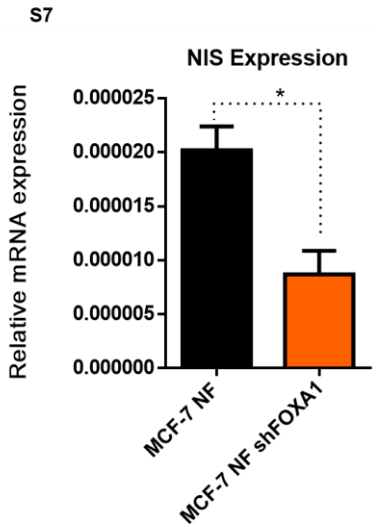

Supplementary Figure 7: **Depletion of FOXA1 in MCF-7 cells leads to a decrease in NIS transcription.** *Graph showing relative mRNA expression of NIS. Error bar indicates SEM. * indicates p≤0.05.*

S8

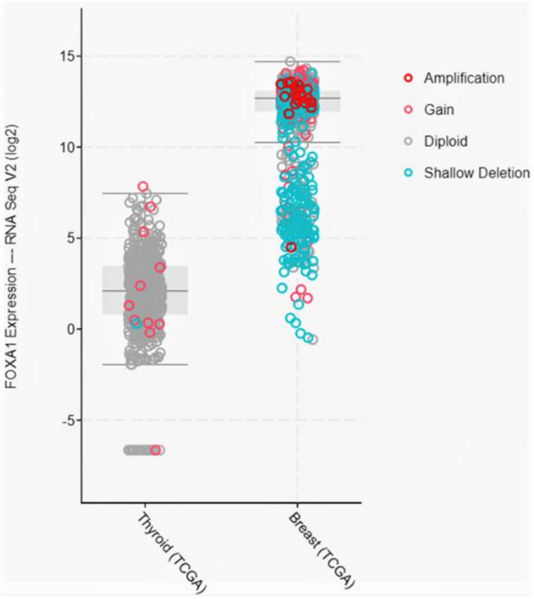

Supplementary Figure 8: **FOXA1 expression is higher in breast cancer patients as compared to thyroid cancer patients.** *Retrospective analysis from TCGA data set showing differential expression of FOXA1 across breast and thyroid cancer patients.*
